# Impact of scaffolding protein TNRC6 paralogs on gene expression and splicing

**DOI:** 10.1261/rna.078709.121

**Published:** 2021-09

**Authors:** Samantha T. Johnson, Yongjun Chu, Jing Liu, David R. Corey

**Affiliations:** UT Southwestern Medical Center, Departments of Pharmacology and Biochemistry, Dallas, Texas 75205, USA

**Keywords:** RNA interference, TNRC6, GW182, alternative splicing, Argonaute, RNA sequencing

## Abstract

TNRC6 is a scaffolding protein that bridges interactions between small RNAs, argonaute (AGO) protein, and effector proteins to control gene expression. There are three paralogs in mammalian cells, *TNRC6A*, *TNRC6B*, and *TNRC6C*. These paralogs have ∼40% amino acid sequence identity and the extent of their unique or redundant functions is unclear. Here, we use knockout cell lines, enhanced crosslinking immunoprecipitation (eCLIP), and high-throughput RNA sequencing (RNA-seq) to explore the roles of TNRC6 paralogs in RNA-mediated control of gene expression. We find that the paralogs are largely functionally redundant and changes in levels of gene expression are well-correlated with those observed in *AGO* knockout cell lines. Splicing changes observed in *AGO* knockout cell lines are also observed in *TNRC6* knockout cells. These data further define the roles of the TNRC6 isoforms as part of the RNA interference (RNAi) machinery.

## INTRODUCTION

Scaffolding proteins play critical roles in biology by bringing proteins with diverse functions into proximity (Shaw and Filbert 2009). Their ability to guide the formation of complexes increases the effective concentrations of proteins and nucleic acids relative to one another, allowing for more efficient activities inside cells. One important example of scaffolding proteins is the GW182 family (Eystathioy et al. 2002) which plays a critical role facilitating the regulation of gene expression during RNA interference (RNAi) (Baillat and Shiekhattar 2009; Takimoto et al. 2009; Niaz and Hussain 2018).

**FIGURE 1.**
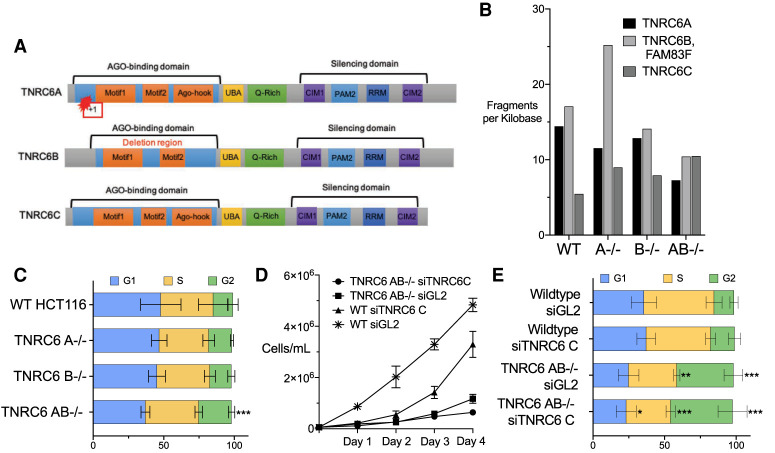
Effect of loss of TNRC6A, TNRC6B, or TNRC6C expression on cell cycle and cell proliferation. (*A*) Diagram of TNRC6A, TNRC6B, and TNRC6C proteins, with known motifs, knockout mutations, and deletions. (*B*) Bar graph of TNRC6 paralog's fragments per kilobase values obtained from whole-cell RNA sequencing of knockout cell lines. TNRC6B and FAM83F are overlapping genes. (*C*) Percentage of cells in each stage of the cell cycle. (*D*) Growth curve for cell lines transfected with anti-TNRC6C siRNA. (*E*) Percentage of cells in each stage of the cell cycle for transfected cell lines wild-type after transfection with siTNRC6C, or control duplex siGL2. (*) *P* > 0.05; (**) *P* > 0.01. (***) *P* > 0.001.

In vertebrates, there are three GW182 protein paralogs, also known as trinucleotide repeat containing protein 6A (TNRC6A), TNRC6B, and TNRC6C. These are multidomain proteins consisting of an argonaute (AGO) binding domain that can bind up to three AGO protein paralogs (Nishi et al. 2013; Pfaff et al. 2013; Elkayam et al. 2017), CCR4-NOT interacting motif (CIM) (Chekulaeva et al. 2011; Fabian et al. 2011), an ubiquitin associated-like (UBL) domain (Nishi et al. 2013), a glutamine rich domain (Q-rich) (Baillat and Shiekhattar 2009), a PABP-interacting motif 2 (PAM2) (Fabian et al. 2009; Lazzaretti et al. 2009), and an RNA recognition motif (RRM) (Fig. 1A; Eulalio et al. 2009).

For RNA interference (RNAi), TNRC6 plays a critical bridging role. AGO protein binds miRNA guide strands and the miRNA:AGO complex associates with complementary target RNA sequences. TNRC6 binds to AGO through the one of two (in TNRC6B) or three (TNRC6A or TNRC6C) motifs in the AGO binding domain (Fig. 1A; Elkayam et al. 2017). The ability of TNRC6 to bind multiple AGO proteins permits enhanced association through cooperative binding between two or three AGO:miRNA complexes (Broderick et al. 2011; Gebert and MacRae 2019; Briskin et al. 2020). Mass spectrometry has identified other accessory proteins binding to TNRC6 domains that may contribute to the control of gene expression (Hicks et al. 2017; Suzawa et al. 2017; Sarshad et al. 2018). The best known TNRC6 interactor is the CCR4-NOT complex, which is responsible for translation repression during RNAi (Behm-Ansmant et al. 2006; Fabian et al. 2011; Collart 2016) but was initially discovered as a regulator of gene transcription (Albert et al. 2000).

This partnership between AGO and TNRC6 proteins is central to understanding how RNAi governs gene expression. Here, we use *TNRC6* knockout and knockdown cells deficient in *TNRC6A*, *TNRC6B*, and *TNRC6C* expression in combination with enhanced crosslinking immunoprecipitation (eCLIP) (Van Nostrand et al. 2016) and RNA sequencing (RNA-seq) to investigate the potential for unique and redundant function for the TNRC6 paralogs during RNAi. We find that the TNRC6 paralogs are largely redundant and that effects on gene expression are remarkably consistent to those observed when AGO proteins are knocked out.

## RESULTS

### Experimental design: TNRC6 knockout and knockdown cells

We have previously described *TNRC6A*^−/−^, *TNRC6B*^−/−^, and *TNRC6AB*^−/−^ knockout cell lines (Liu et al. 2019). HCT116 colorectal cancer cells were chosen as a parental line because they are diploid, which facilitates knocking out multiple genes simultaneously. We felt that knocking out multiple TNRC6 variants might be important because of the potential for redundant function. The use of HCT116 as a parental line also allows us to compare the TNRC6 lines directly with AGO knockout cell lines that were previously created from HCT116 cells (Chu et al. 2020, 2021).

The knockout of the TNRC6A protein was confirmed by western blot analysis (Supplemental Fig. 1A). We did not possess an adequate antibody to detect TNRC6B protein and knockout of TNRCB protein expression was confirmed by mass spectrometry (Liu et al. 2019). The knockout of *TNRC6A* was due to a point mutation that produced a frameshift, while the knockout of *TNRC6B* was achieved by a 95,481 bp deletion (Fig. 1A). *TNRC6A* and *TNRC6B* are the most abundant paralogs in HCT116 cells. *TNRC6C* is up-regulated in the *TNRC6 A*^−/−^ and *TNRC6 B*^−/−^ cell lines, suggesting the potential to compensate for their loss (Fig. 1B).

*TNRC6A*^−/−^ or *TNRC6B*^−/−^ single knockout cells grew slower than wild cells (Liu et al. 2019). *TNRC6AB*^−/−^ double knockout cells were the slowest proliferating. We could not obtain a *TNRC6ABC*^−/−^ triple mutant, consistent with residual TNRC6 function being necessary for cell growth. To examine the effect of loss of TNRC6C protein expression we used a pool of anti-*TNRC6C* duplex RNAs that knocked down >90% of *TNRC6C* expression (Supplemental Fig. 1B).

### Effect of TNRC6 paralog expression on cell cycle

To further evaluate the impact of the TNRC6 paralogs on cell proliferation, we examined the consequences of *TNRC6* knockout on cell cycle. The *TNRC6A*^−/−^ and *TNRC6B*^−/−^ knockout cell lines showed no significant changes throughout the cell cycle (Fig. 1C). Double knockout *TNRC6AB*^−/−^ cells had a significant increase in G2 phase (Fig. 1C). The change in cell cycle stage for the double knockout cells is consistent with the reduced cell growth seen in the *TNRC6AB*^−/−^ cells (Liu et al. 2019).

Because we could not obtain triple knockout *TNRC6ABC*^−/−^ cells, we used an siRNA pool to knock down *TNRC6C* expression (Supplemental Fig. 1B). As a control, we also transfected a noncomplementary duplex RNA, siGL2, into both wild-type HCT116 and *TNRC6AB*^−/−^ double knockout cells. We observed that wild-type cells transfected with control duplex siGL2 grew faster than cells transfected with the siTNRC6C pool (Fig. 1D). For *TNRC6AB*^−/−^ double knockout cells, growth was decreased regardless of whether in the siGL2 or the siTNRC6C pool was introduced into cells. Reduced cell growth in the TNRC6AB^−/−^ cells, observed regardless of the knockdown of *TNRC6C* expression, may be related to the general decrease in fitness for these cells. Cell growth may be sensitive to addition of lipid/oligonucleotide complexes rather than being directly impacted by impairment of RNAi pathways.

When *TNRC6C* expression alone is knocked down using an siRNA pool, the cell cycle does not change significantly relative to addition of the control duplex (Fig. 1E). When both *TNRC6A* and *TNRC6B* expression are knocked out, the addition of either control or anti-*TNRC6C* duplex RNA in complex with lipid dramatically alters the cell cycle. Transfection with lipid can stress cells. These data suggest that the loss of most functions of the three TNRC6 paralogs may have a bigger impact when the cells are challenged by environmental change, an outcome similar to the cell growth impairment observed above.

### Impact of TNRC6 knockout/knockdown on gene expression

**FIGURE 2.**
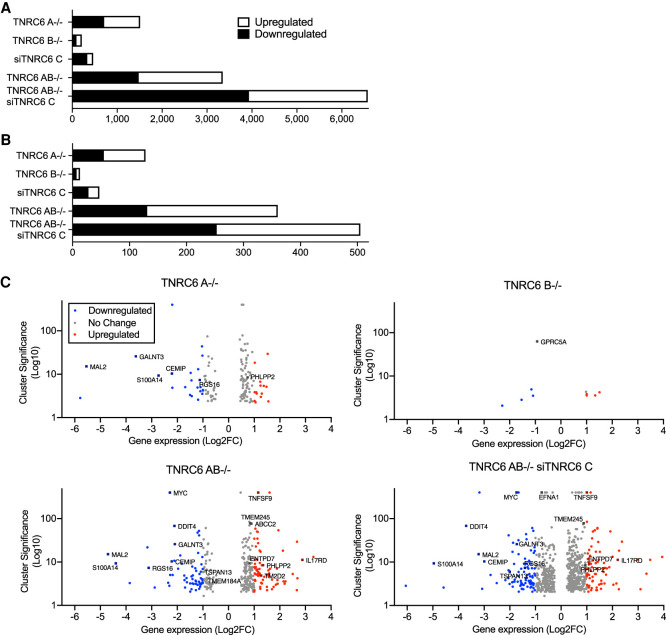
Association of AGO2 protein binding and gene expression in *TNRC6* knockout cells. (*A*) Total number of significantly up- or down-regulated genes in knockout cell lines. (*B*) Total number of significantly up- or down-regulated genes in knockout cell lines that overlap with AGO2 binding sites in coding sequences (CDS) and in the 3′ untranslated regions (3′UTR). (*C*) Volcano plots of gene expression in *TNRC6* knockout cell lines.

We used RNA sequencing (RNA-seq) of whole cells to evaluate the impact of knocking down or knocking out *TNRC6A*, *TNRC6B*, and *TNRC6C* on overall gene expression (Fig. 2). Read depth was consistent across all samples. Knocking out *TNRC6A* had a greater effect on gene expression than knocking out *TNRC6B* or knocking down *TNRC6C* (Fig. 2A). The *TNRC6AB*^−/−^ double knockout has a larger effect than the single knockout cell lines (Fig. 2A). The greatest impact on gene expression was observed for the combination of *TNRC6AB*^−/−^ knockout and *TNRC6C* siRNA-mediated knockdown (Fig. 2A).

We have previously used enhanced crosslinking immunoprecipitation (eCLIP) (Van Nostrand et al. 2016) to identify locations within the transcriptome where AGO2 binds (Chu et al. 2020). Genes that host these sites are candidates for regulating gene expression because significant AGO2 binding is thought to be correlated with recognition of miRNAs (Lewis et al. 2003; Friedman et al. 2009; Gebert and MacRae 2019; Chu et al. 2020; Eisen et al. 2020). These regions were identified by clusters of RNA-seq reads that were not present or not significantly enriched when compared to parallel experiments using AGO2 knockout cells or a size-matched input sample.

The standard mechanism for endogenous miRNA regulation in mammalian cells suggests that regulation is through interactions within the 3′-untranslated region (3′-UTR). Therefore, we identified mRNAs that possessed read clusters within their 3′-UTRs. We then measured the effect of TNRC6 loss on the expression of these genes in knockout versus wild-type cells (Fig. 2B).

In all cell lines examined, only a small fraction of gene expression changes (Fig. 2A) were associated with significant AGO2 binding clusters (Fig. 2B). As we had observed for overall gene expression, the number of genes with altered expression was less in single knockout cells, greater in double knockout cells, and greatest in the *TNRC6AB*^−/−^ siTNRC6C cell line. Once again, the *TNRC6A*^−/−^ knockout had a bigger effect on expression than the *TNRC6B*^−/−^ knockout or siTNRC6C knockdown.

A standard assumption of miRNA action is that binding of an AGO:miRNA complex within the 3′-UTR will repress gene expression (Friedman et al. 2009; Guo et al. 2010; Jonas and Izaurralde 2015; Gosline et al. 2016; Gebert and MacRae 2019) and that knocking out AGO variants should increase gene expression. While reducing expression of RNAi factors like the TNRC6 variants would be expected to produce a complex mix of gene expression changes—some direct and some indirect—genes that associate with AGO2 would be expected to be de-repressed when critical RNAi factors are knocked out or knocked down.

We observed, however, that regardless of whether we examine the expression of all genes (Fig. 2A) or only genes with AGO binding clusters (Fig. 2B), that similar numbers of genes were associated with up- and down-regulation. These data indicate that there is no simple correlation between AGO2 occupancy and up- or down-regulation of a transcript.

We then used volcano plots to visualize cluster significance and fold change of individual genes (Fig. 2C). Once again, the *TNRC6 A*^−/−^ cell line showed more profound changes than *TNRC6 B*^−/−^ cells. The *TNRC6 AB*^−/−^ cells or *TNRC6 AB*^−/−^ siC cells showed much greater effects than the single gene knockout cells, both in terms of the number of genes changed and the magnitude of gene expression changes. Cluster significance, the indication of AGO2 occupancy, was not associated primarily with up or down regulation regardless of which knockout cell line is examined. We also plotted the correlation of all significant genes with and without AGO2 binding clusters in TNRC6 AB^−/−^ siC and AGO123^−/−^. We found a significant correlation above 0.65 (Supplemental Fig. 2), supporting the conclusion that they participate in a common pathway.

### Comparing the impact of AGO and TNRC6 knockouts on global gene expression

**FIGURE 3.**
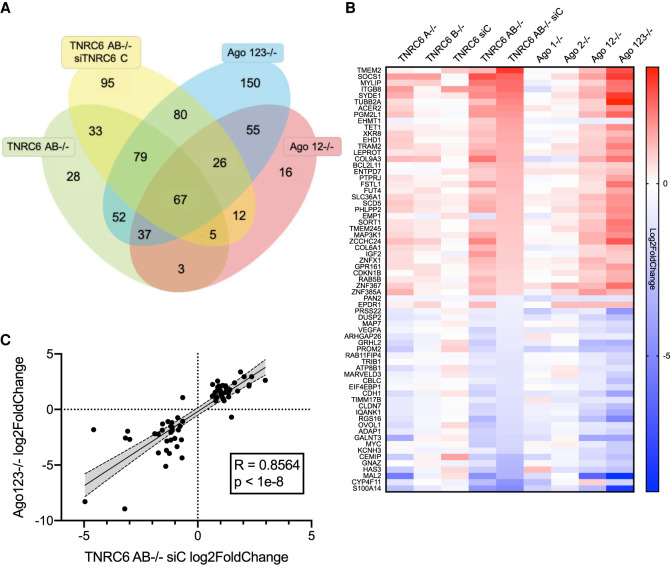
Consistent variation for AGO2 protein binding cluster's gene expression changes in *TNRC6* and *AGO* knockout cells. (*A*) Venn diagram showing the overlap of gene expression changes associated with AGO2 binding within 3′-UTRs. AGO2 binding was determined by eCLIP as described (Chu et al. 2020) and was required for inclusion. (*B*) Heatmap showing gene expression changes (Log_2_FoldChange) shared by *TNRC6* and *AGO* knockout cell lines. (*C*) Correlation plot of the 67 overlapping genes shown in *B* of AGO 123^−/−^ and TNRC6 AB^−/−^ siC log_2_FoldChanges. Shaded region is 95% confidence band.

The TNRC6 protein paralogs are important binding partners for AGO proteins (Kalantari et al. 2016b; Hicks et al. 2017). The scaffolding domains of TNRC6 facilitate the recruitment of effector proteins for mRNA degradation (Piao et al. 2010; Chekulaeva et al. 2011; Fabian et al. 2011; Hicks et al. 2017) or transcriptional activation (Hicks et al. 2017; Liu et al. 2018, 2019). Because of the partnership between AGO and TNRC6 we hypothesized that many gene expression changes would be shared between *TNRC6* and *AGO* knockout cells and those genes would be the best candidates as endogenous control points for regulation by miRNAs. It is also possible, however, that AGO and TNRC6 proteins may play independent roles. To evaluate these hypotheses and identify candidate genes, we compared the impact on gene expression of knocking out AGO and TNRC6 proteins (Fig. 3).

We focused on genes that had significant AGO2-binding clusters within their 3′-UTRs (Chu et al. 2020) and compared the gene expression changes in *TNRC6AB*^−/−^, *TNRC6AB*^−/−^ siTNRC6C, *AGO12*^−/−^, and *AGO123*^−/−^ cells relative to wild-type HCT116 cells (Fig. 3A). When examining large data sets, it is important to prioritize outputs. We reasoned that genes showing expression changes in multiple cell lines would be the best candidates for physiologically relevant gene regulation. Gene expression changes due to experimental noise or artefacts from RNA-seq are least likely when the changes occur in multiple cell lines. We recognize that these stringent criteria may overlook some candidates, but they facilitate focusing on a manageable number of genes for further analysis.

We identified 67 genes with AGO2 binding clusters and significantly changed gene expression (FDR < 0.05, −0.6>Log_2_ Fold Change > 0.6) that were shared in all of the four cell lines (Fig. 3A). These 67 genes included examples of both up- and down-regulation, with 36 genes increasing expression and 31 genes with reduced expression.

We then used heat map analysis to sort these genes according to altered gene expression and to extend the comparison to our *AGO* knockout cell lines (Fig. 3B). Of the nine cell lines examined, the siTNRC6C knockdown cells showed the least change and no obvious correlation for up- or down-regulation. Of the remaining *TNRC6* knockout cell lines, gene expression changes were weakest in *TNRC6B*^−/−^ cells, stronger in *TNRC6A*^−/−^ cells, and strongest in the *TNRC6AB*^−/−^ and *TNRC6AB*^−/−^ siC cells—a comparison reminiscent of our data for cell proliferation, cell cycle analysis, and global gene expression. For the eight knockout cell lines, genes that were up-regulated in *AGO* knockout cells tended to be up-regulated in *TNRC6* knockout cells, while genes that were down-regulated in *AGO* knockout cells showed similar down-regulation in the engineered *TNRC6* cells (Fig. 3B).

We examined the correlation of the gene expression changes in the *TNRC6AB^−/−^ siC* and *AGO123*^−/−^ cells for the 67 genes with AGO2 binding clusters and significantly changed gene expression. These shared genes had a correlation factor of 0.86, indicating correlation of between *TNRC6* and *AGO* knockout cell lines (Fig. 3C). This correlation is consistent with TNRC6 and AGO proteins playing critical roles in common gene regulatory pathways, presumably the RNAi pathway.

**FIGURE 4.**
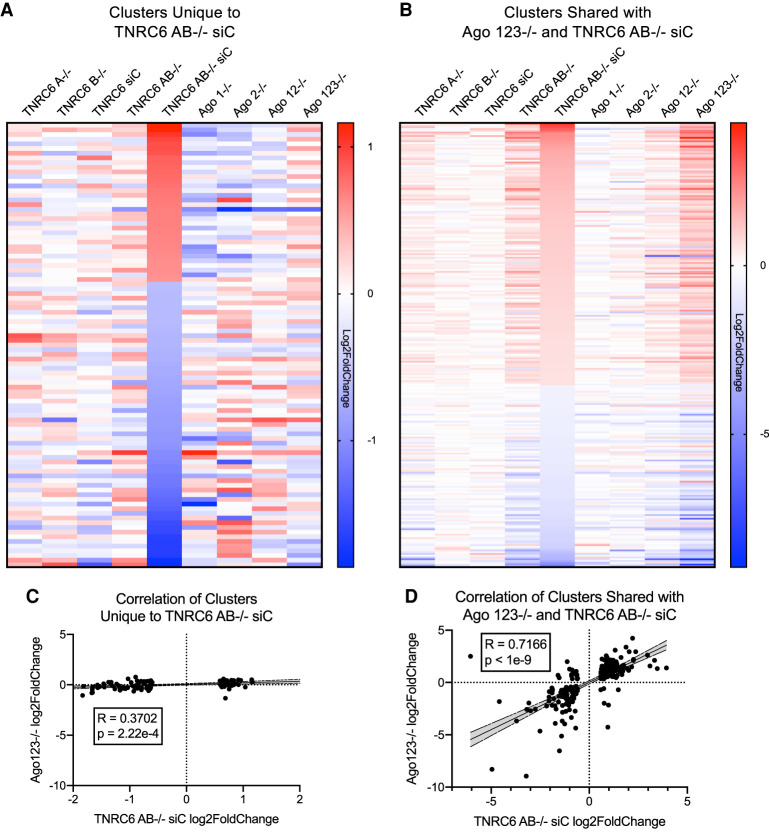
Comparison of gene expression in *TNRC6* and *AGO* knockout cells. (*A*) Heatmap of Log_2_FoldChange gene expression changes of 95 genes with AGO2 protein binding clusters that change significantly only in the *TNRC6AB*^−/−^ siTNRC6C cells. (*B*) Heatmap of Log_2_FoldChange gene expression changes of 252 genes with AGO2 clusters that change significantly in *TNRC6 AB^−/−^ siTNRC6C* and *AGO123*^−/−^ cells (not including the 67 genes previously shown in Fig. 3B). (*C*) Correlation plot of the 95 genes unique to TNRC6 AB^−/−^ siC shown in *A* of AGO 123^−/−^ and TNRC6 AB^−/−^ siC log2FoldChanges. Shaded region is 95% confidence band. (*D*) Correlation plot of the 252 genes changed in AGO123^−/−^ and TNRC6 AB^−/−^ siC in shown in *B* of AGO 123^−/−^ and TNRC6 AB^−/−^ siC log_2_FoldChanges. Shaded region is 95% confidence band.

For comparison, we also examined gene expression changes in other overlapping cohorts of knockout cells. For example, the 95 genes that were changed in the *TNRC6 AB*^−/−^ siTNRC6C cell lines, but no other cell line (Fig. 3A), did not show similar gene expression trends relative to the other cell types (Fig. 4A). The correlation factor for these 95 genes was less than 0.5, indicating no correlation (Fig. 4C). This result supports the conclusion that these gene expression changes are unrelated to perturbation of the RNAi pathway. Conversely, 252 changes shared between the *AGO123*^−/−^ and the *TNRC6AB*^−/−^ siTNRC6C cells mostly overlap (Fig. 4B) with a correlation of 0.72 (Fig. 4D), suggesting that these genes are more likely to be regulated by RNAi and is consistent with correlation of the smaller subset of 67 genes examined in Figure 3B.

When evaluating CLIP-seq data, it is essential to view the primary data to evaluate the characteristics of each cluster of reads to ensure the quality of the data and identify different classes of read cluster. In this case, we visually inspected the top 200 most significant clusters associated with 3′-UTR RNA. Previously, we had focused our experimental validation of gene expression in AGO knockout cells on 22 representative genes with distinctive and unambiguous AGO2-binding clusters (Chu et al. 2020). These genes were chosen to represent differing species of highly significant clusters (single clusters versus multiple closely space clusters), and both up- and down-regulated genes. These clusters were both significant relative to input or samples from AGO2 knockout cells and were composed of relatively large numbers of sequencing reads. The cluster sites contained seed multiple sequence complementarity matches with well-expressed miRNAs.

**FIGURE 5.**
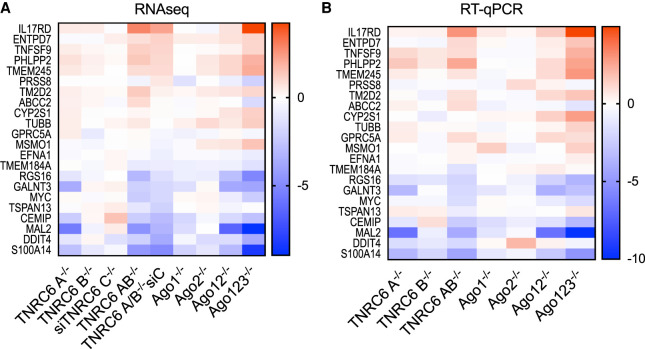
Consistent variation of gene expression in cells with highly ranked AGO2 protein binding clusters. (*A*) Heatmap of gene expression changes from *TNRC6* knockout and *AGO* knockout cell lines from RNA sequencing for 22 cluster genes examined in Chu et al. (2020). (*B*) Heatmap of gene expression changes from *TNRC6* knockout and *AGO* knockout cell lines from RT-qPCR for 22 cluster genes examined in Chu et al. (2020).

We compared the gene expression of these 22 chosen genes in our nine knockout or knockout/knockdown data sets. Similar to the result observed with our 67 gene overlapping cohort (Fig. 3), the selected 22 genes showed a similar rank order of gene expression change regardless of whether AGO or TNRC6 protein variants were being knocked out (Fig. 5A). As observed for the 67 gene cohort, the siTNRC6C knockdown cells did not trend with the other cell lines, suggesting that the *TNRC6C* knockdown had the least impact on cells. These data demonstrate that the broad trends correlating the impact of gene expression of TNRC6 and AGO knockdown also apply to genes with the strongest AGO2 association detected by eCLIP.

Quantitative PCR (qPCR) was performed to validate the RNA-seq data (Fig. 5B). Measurement of RNA samples from each cell line confirmed the trends observed in the RNA-seq data. Further, correlation plots of these 22 selected genes show correlations greater than 0.8 in both RNA-seq and qPCR data, supporting the conclusion from visual inspection that the impact on this subset of genes from knocking out *AGO* and *TNRC6* genes is significantly correlated (Supplemental Fig. 3). These data suggest that the trends correlating *AGO* or *TNRC6* knockout remain similar regardless of the shape of the RNA read cluster detected by eCLIP and RNA-seq.

### Impact of AGO and TNRC6 variants on alternative splicing

RNAi has been suggested to have the potential to directly regulate splicing (Allo et al. 2009; Liu et al. 2012, 2015; Fuchs et al. 2021). In a related study, we have examined the impact of knocking out AGO variants on gene splicing (Chu et al. 2021). eCLIP was used to identify sites of AGO2 binding within intronic RNA. We now examine the impact of knocking out TNRC6 paralogs on splicing to assess the involvement of TNRC6 on the regulation of endogenous splicing by miRNAs.

**FIGURE 6.**
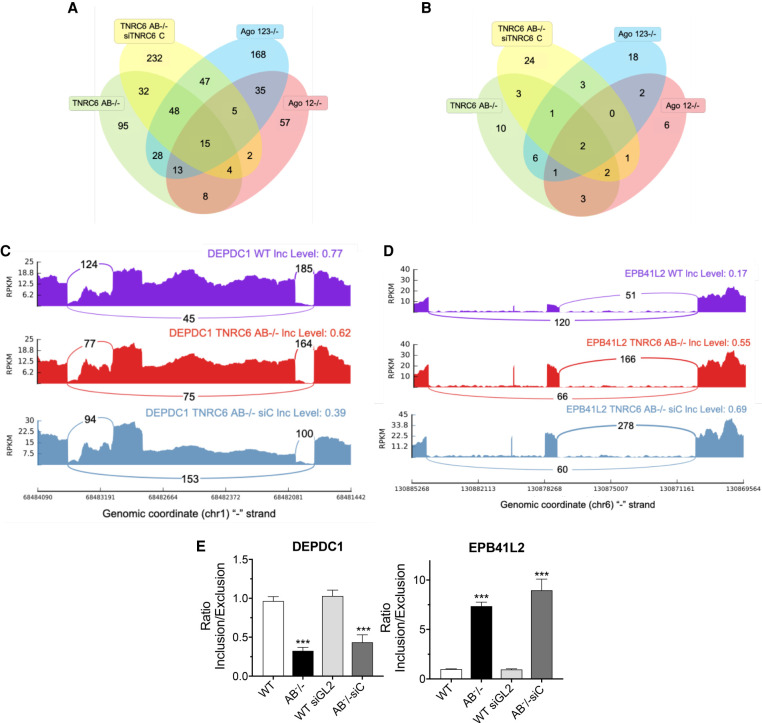
Changes in alternative splicing in *TNRC6* knockout cell lines. (*A*) Venn diagram of skipped exon splicing events. (*B*) Venn diagram of skipped exon splicing events located near AGO2 binding clusters. (*C*,*D*) Sashimi plots for genes *DEPDC1* and *EPB41L2* that overlap between *TNRC6 AB*^−/−^ and *TNRC6 AB^−/−^ siC* RNA-seq data sets in *B*. (*E*) qPCR validation of skipped exon events in *TNRC6 A/B* knockout and *TNRC6 A/BKO/siC* cells. Error bars represent standard deviation (SD). (*) *P* < 0.05; (**) *P* < 0.01; (***) *P* < 0.001 compared with control cell by two tailed *t*-test.

We evaluated the changes in splicing observed in our knockout cell lines. Venn diagrams were used to visualize all skipped exon splicing events that were changed in *AGO123*^−/−^, *AGO12*^−/−^, *TNRC6AB*^−/−^, and *TNRC6AB*^−/−^ siTNRC6C cell lines (Fig. 6A). Changes that were observed in all four lines were awarded the highest priority for analysis because we reasoned that shared events would be most likely to have physiological relevance.

We found that fifteen skipped exon splicing events are shared between the four cell lines (Fig. 6A). Of those fifteen, only two genes (*DEPDC1* and *EPB41L2*) had significant AGO2 binding clusters located within affected introns (Fig. 6B). Visual inspection of the sashimi plots for *DEPDC1* showed an increase in exon skipping in the *TNRC6AB*^−/−^ and *TNRC6AB*^−/−^siC cell lines relative to wild-type (Fig. 6C), while *EPB41L2* sashimi plots showed a decrease in exon skipping for the knockout cell lines (Fig. 6D). The splicing changes seen in the RNA-seq data for *DEPDC1* and *EPB41L* were validated by qPCR (Fig. 6E). Splicing changes for *EPB41L2* were further validated by PCR (Supplemental Fig. 4).

**FIGURE 7.**
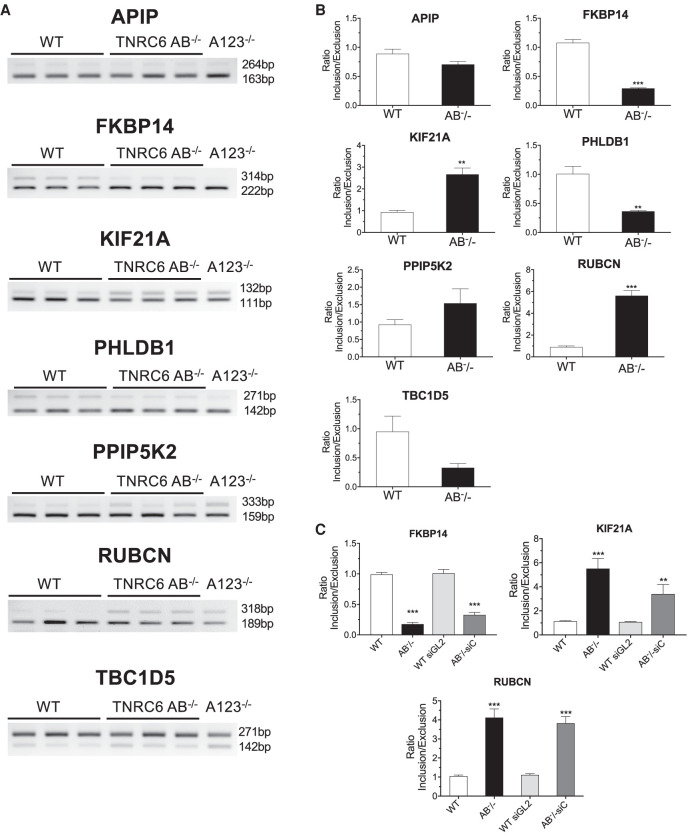
Validating the effect of *TNRC6* knockouts on alternative splicing. (*A*) Semiquantitative PCR validation of skipped exon events in *TNRC6 A/B* knockout cells. (*B*) Quantitation of data shown in in part *A*. (*C*) QPCR validation of skipped exon events in *TNRC6 A/B* knockout and *TNRC6 A/B* knockout/*siCTNRC6* knockdown cells. Error bars represent standard deviation (SD). (*) *P* < 0.05; (**) *P* < 0.01; (***) *P* < 0.001 compared with control cell by two tailed *t*-test.

Our criteria for splicing changes that occur in all cell lines is restrictive. Intronic RNA is recovered at relatively low amounts and we recognized that our criteria might cause us to overlook some candidates. We chose, therefore, to examine the impact of TNRC6 knockouts on seven genes that our laboratory had already evaluated for splicing changes due to knockout of AGO proteins (Fig. 7; Chu et al. 2021). These seven genes were chosen because they had at least one AGO2 cluster near a significant splicing event locus within an intron and had a miRNA candidate complementary to a sequence within the AGO2 cluster.

Of the seven genes analyzed, our RNA-seq data for TNRC6 knockout cells showed significant changes in alternative splicing for five genes in either the *TNRC6 AB*^−/−^ or *TNRC6AB*^−/−^ siC cells. We evaluated splicing changes by reverse transcriptase PCR (Fig. 7A,B). For five genes, *FKBP14*, *KIF21A*, *PHLDB1*, *RUBCN*, and *TBC1D5*, RT-PCR data confirmed significant splicing changes in both *TNRC6 AB*^−/−^ and *AGO 123*^−/−^ cells (Fig. 7B).

For *FKBP14, KIF21A,* and *RUBCN*, qPCR data confirmed splicing changes in *TNRC6 AB*^−/−^ and *TNRC6AB*^−/−^ siC (Fig. 7C). RT-PCR further confirmed splicing changes for *FKBP14* and *KIF21A* in the TNRC6AB^−/−^ siC cell line (Supplemental Fig. 5). Two genes, *APIP* and *PPIP5K2*, did not have significant detected changes in RNA-seq data and we also did not observe significant changes by PCR (Fig. 7A,B). Side by side quantitation of data from TNRC6 and AGO knockout cell lines confirmed the close correlation for five of seven genes between results from the two sets of knockout cells (Supplemental Fig. 6).

Two genes, *RUBCN* and *FKBP14*, that showed splicing changes in the TNRC6 knockout cells were examined in more detail in Chu et al. (2021). Their splicing was shown to be modulated by miRNA mimics or anti-miRNA oligonucleotides that target the site for AGO2 association determined through eCLIP. These data reinforce the conclusion the suggestion that the genes may be targets for endogenous small RNAs.

## DISCUSSION

### TNRC6 paralogs and RNAi

The three human TNRC6 protein paralogs, TNRC6A, TNRC6B, and TNRC6C, play important roles in RNAi (Baillat and Shiekhattar 2009; Niaz and Hussain 2018). The complex between a small RNA and AGO proteins recognizes the target sequences within cellular RNA, while the three multidomain TNRC6 paralogs bind to AGO and act as scaffolds to promote association with proteins that modulate function. While the three protein paralogs are ∼40% identical, their potential for unique or redundant activities has not been determined. Neither has the extent to which their impact on endogenous gene expression overlaps the impact of AGO proteins.

We had previously investigated the impact of knocking out TNRC6 paralogs on the ability of synthetic duplex RNAs to control translation and splicing (Liu et al. 2019). In those studies, we found that knocking out TNRC6 expression did not affect inhibition of translation or splicing by fully or highly complementary synthetic duplex RNAs but did reverse the action of synthetic miRNA mimics. Here we use knockout cell lines and an efficient siRNA pool that reduces TNRC6C expression to analyze the role of TNRC6 expression on global gene expression.

### Redundancy or independence: roles for TNRC6 paralogs

We had previously used knockout cells to demonstrate that the loss of all three TNRC6 paralogs was required to affect gene activation by synthetic duplex RNAs (Liu et al. 2019). Consistent with this observation, we now observe knocking out TNRC6A or TNRC6B alone has no significant effect on the cell cycle (Fig. 1C). Larger impacts on cell cycle were observed in the *TNRC6 AB*^−/−^ double knockout cell line or *TNRC6 AB*^−/−^ siTNRC6C cells (Fig. 1C,E). Analysis of gene expression or alternative splicing revealed similar results. Blocking expression of two or three TNRC6 paralogs affected expression (Fig. 2) or alternative splicing (Figs. 6, 7) of many more genes than did blocking expression of TNRC6A, TNRC6B, or TNRC6C alone. The conclusion that TNRC6 paralogs have largely redundant functions regulating endogenous gene expression is consistent with our previous observation of redundant function when modulating the effects of designed synthetic RNAs.

While the single knockouts showed little change relative to double knockout cells, we did observe substantially larger number of genes with altered expression in the *TNRC6 A*^−/−^ cells than in *TNRC6 B*^−/−^ cells. These data may indicate that that TNRC6A plays a unique role in regulating expression of a subset of genes.

### How do TNRC6 paralogs affect regulation by RNAi?

We have used eCLIP to identify sites for AGO2 binding within cytoplasmic (Chu et al. 2020) and nuclear (Chu et al. 2021) RNA and used these data to understand how AGO binding correlates with gene expression and splicing at these sites. One conclusion from these studies was that, contrary to the standard expectation that AGO2 binding within a 3′-UTR should be associated with gene repression, we observed that genes with significant association to AGO2 showed up- and down-regulation upon gene knockouts.

Here we report that the effects of knocking out TNRC6 paralogs on the expression of genes with significant AGO2 binding sites yield remarkably similar results (Fig. 3). We can make several conclusions from these data: (i) TNRC6 proteins are largely redundant, although knockout of TNRC6 A has the largest effect. As additional TNRC6 paralogs are removed, effects become greater; (ii) the similarity of up- and down-regulated genes reveals the remarkable extent to which AGO and TNRC6 proteins function as partners to control gene expression; (iii) as with AGO knockout cells, knocking out TNRC6 paralogs has an unpredictable effect on gene expression even for genes that possess experimentally determined AGO2 binding sites within their 3′-UTRs. The fact that AGO2 has a significant association with a 3′-UTR cannot be assumed to lead to gene up-regulation when AGO or TNRC6 proteins are removed from cells.

We have not resolved whether the changes in gene expression we observe are due to direct effects of miRNAs binding to sites where AGO2 association is detected or indirect effects. Indeed, the difficulty of assigning a direct effect to a particular site of AGO2 association is a primary finding of our studies. The expression of 6000 genes change (Fig. 2A) upon knockout or knockdown of the *TRNC6* variants, suggesting a large capacity for indirect change—either repression or activation. Even for genes where expression changes occur at genes with significant AGO2 binding within 3′-UTRs, the simplest conclusion based on the potential for indirect effects is that a cause-and-effect relationship between AGO binding and activation/repression of gene expression cannot be assumed. Further experimental validation is necessary for each candidate.

Because of the lack of predictable correlation with gene repression or up-regulation, such studies are not straightforward and will be a subsequent focus of research. It is clear from the data, however, that the genes we identify are being controlled by a common RNAi axis that requires expression of both AGO and TNRC6. The fact that we observe both increased and decreased expression at genes with AGO2 association within their 3′-untranslated regions supports our previous conclusion from AGO knockout cells that AGO2 occupancy is not sufficient to infer repression of a target transcript and emphasize the complexity of RNAi function.

### Impact of TNRC6 on alternative splicing

While RNAi is often assumed to be a cytoplasmic mechanism in mammalian cells (Zeng and Cullen 2002), RNAi protein factors and miRNAs also exist in cell nuclei (Gagnon et al. 2014). Functional evidence showing robust control of transcription and splicing by synthetic duplex RNAs (Allo et al. 2009; Liu et al. 2012, 2015; Kalantari et al. 2016a) has suggested the potential for nuclear RNAi to be a natural regulatory mechanism, but persuasive experimental evidence for control of endogenous transcription or splicing has been elusive. In addition, previous studies using synthetic RNAs had shown that TNRC6 is not required for highly complementary synthetic small RNA to influence differential splicing (Liu et al. 2018).

In Chu et al. (2021), we investigate the impact of endogenous miRNAs and RNAi on alternative splicing. We identify sites of AGO2 binding using the same eCLIP data set used here and correlate AGO2 binding with changes in splicing upon knocking out AGO1, AGO2, and AGO3. We observe changes in splicing and show that splicing can be manipulated by synthetic miRNAs or anti-miRs designed based on predictions of miRNAs that target sites identified by eCLIP.

We now show that knocking TNRC6 variants also affect alternative splicing. As with our AGO data sets, only a relatively small number of candidate splicing events are identified. However, of this small number, there was substantial overlap between our TNRC6 and AGO data, supporting belief they are due to a common RNAi-related pathway. The expression of two of the genes with splicing changes in both *AGO*^−/−^ and *TNRC6*^−/−^ data sets, *RUBCN* and *FKBP14*, could be manipulated by miRNA mimics or antimiRs designed to predicted target sites.

These data suggest that miRNAs have the potential to control endogenous splicing. We had previously reported that duplex RNAs that control splicing did not require expression of TNRC6 (Liu et al. 2019). These RNAs, however, were either fully or almost fully complementary to their target sites within intronic RNA. The scaffolding function of TNRC6 acts to bridge AGO proteins, increasing the cooperativity of binding and allowing imperfectly paired miRNAs to associate with target sequences more tightly (Elkayam et al. 2017). It is possible that, while TNRC6 is not necessary for recognition of highly complementary duplex RNAs, it is necessary for the activity of mixtures of imperfectly complementary miRNAs that act in concert to control endogenous gene expression.

Our data also suggest that AGO and TNRC6 affect the splicing of a relatively small subset of proteins. This outcome is consistent with the conclusion that RNA-mediated regulation of splicing is a minor regulatory mechanism in HCT116 cells. RNAi-mediated regulation of splicing may also be more pervasive in other cell types, cells grown under more demanding environmental conditions, during cell development, or in cells involved in disease. Alternatively, intronic RNA is present at relatively low steady state levels (Clement et al. 1999; Mortazavi et al. 2008) and therefore less detectable. Because of our stringent conditions for identifying candidates for AGO2 binding and splicing change, we may be overlooking genes where RNA-directed modulation of splicing is biologically significant yet undetected by our approach.

### Conclusions

Scaffolding proteins play complex roles bringing other proteins, RNA, or DNA together. The TNRC6 family proteins present a multidomain model for understanding the potential of scaffolding proteins to organize molecular function. (Shaw and Filbert 2009) We observe a strong overlap between the changes in gene expression upon knockout of TNRC6 or AGO proteins, consistent with the partnership of these proteins during RNAi. In our HCT116 model cell line, at least, that partnership does not produce predictable changes in gene expression at sites within target RNAs where AGO2 binds. It is clear from our data that TNRC6 plays an important role in controlling gene expression, but there remains much to learn about how it acts in concert with AGO2 and the possibility that it may play significant roles independent of AGO2.

## MATERIALS AND METHODS

### Cell culture

Wild-type HCT116 cells were obtained from Horizon Discovery. HCT116 cells containing knockout modifications to the *TNRC6A, TNRC6B,* and *TNRC6A & TNRCB* genes were purchased from GenScript. All cell lines were cultured in McCoy's 5A medium (Sigma-Aldrich) supplemented with 10% FBS (Sigma-Aldrich) in 37°C 5% CO_2_. For cell counting, cells were mixed together with equal volumes of trypan blue (Sigma) and were counted using cell counter (TC20 Automated Cell Counter; Bio-Rad).

### Transfections

All transfections used Lipofectamine RNAi MAX (Invitrogen). For transfections, cells were seeded into six-well plates at 150,000 cells per well for wild-type, *TNRC6 A*^−/−^ and *TNRC6 B^−/−^. TNRC6 AB*^−/−^ cells were seeded at 250,000 cells per well due to the slowed growth rate of these cells. Cells were transfected as described in Liu et al. (2019).

### PI staining and FACS

Cells were harvested at 90% confluency for cell cycle analysis by propidium iodide (PI) staining. Cells were harvested using 1× Trypsin-EDTA (Sigma) and washed with PBS. To fix cells, they were suspended in PBS at a concentration of 2 × 10^6^ cells per mL and added to an equal volume of 100% ethanol while vortexing. Cells were then incubated at −20°C for 24 h to 1 mo. To prepare cells for staining with PI, they were washed three times with PBS to ensure all ethanol was removed. Cells were suspended in staining buffer (0.1% Triton X, 20 µg/mL PI, and 20 µg/mL RNase A) at 2 × 10^6^ cells per mL. Stained cells were incubated at 37°C for 15 min. Cells were then stored at 4°C and protected from the light. Cells were run within 48 h at the UTSW Flow Cytometry Facility on a Caliber. Data was then analyzed using FlowJo software.

### RNA extraction, RNA sequencing, and enhanced crosslinking immunoprecipitation sequencing (eCLIP)

Whole-cell RNA was extracted from cells harvested with trypsin at 90% confluency. RNeasy kit (Qiagen) was used to purify the RNA for whole-cell steady state mRNA sequencing. RNA sequencing was performed by the McDermott Center Next-Generation Sequencing Core at UTSW as described in Chu et al. (2021). RNA sequencing data was analyzed in the same manner as Chu et al. (2021). Methods and analysis for eCLIP were described in Chu et al. (2020).

### qPCR and western blot

Both qPCRs and western blots were performed as described in Liu et al. (2019).

### Splicing analysis by gel electrophoresis and qPCR

Total RNA was extracted from HCT116 wild-type, TNRC6 knockout cells, and treated with DNase I (Worthington Biochemical) at 25°C for 20 min, 75°C for 10 min. Reverse transcription was performed using a high-capacity reverse transcription kit (Applied Biosystems) per the manufacturer's protocol. 2.0 µg of total RNA was used per 20 µL of reaction mixture.

For gel electrophoresis analysis, PCR amplification was performed as following; 95°C 3 min and 95°C 30 sec, 60°C 40 sec, 72°C 30 sec for 35 cycles. The PCR products were separated by 1.5% agarose gel electrophoresis. The bands were quantified by using ImageJ software.

In qPCR analysis for splicing changes by using double strand RNAs and miRNA mimics, PCR was performed on a Biorad CFX384 Real-Time System using iTaq SYBR Green Supermix (BioRad). PCR reactions were done in triplicates at 55°C 2 min, 95°C 3 min and 95°C 20 sec, 60°C 45 sec for 40 cycles in an optical 384-well plate. The expression level was compared between exon included spliceform and exon excluded spliceform. PCR primers were shown in Supplemental Table 3.

## DATA DEPOSITION

All high-throughput sequencing data generated for this study (RNA-seq, eCLIP) have been deposited in the Gene Expression Omnibus under accession number GSE162749.

## SUPPLEMENTAL MATERIAL

Supplemental material is available for this article.

## Supplementary Material

Supplemental Material
