# Supplementary material for: Impact of scaffolding protein TNRC6 paralogs on gene expression and splicing
Source: RNA. 2021 Sep;27(9):1004–16. doi: 10.1261/rna.078709.121 (PMC8370741; doi:10.1261/rna.078709.121)
Supplement: Supplemental Material [file supp_27_9_1004__DC1.html]

Impact of scaffolding protein TNRC6 paralogs on gene expression and splicing — Supplemental Material 

# Impact of scaffolding protein TNRC6 paralogs on gene expression and splicing

## Supplemental Material

- Supplemental\_Figures.pptx
- Supplemental\_Figure\_Legends.docx
