# Supplementary material for: Impact of scaffolding protein TNRC6 paralogs on gene expression and splicing
Source: RNA. 2021 Sep;27(9):1004–16. doi: 10.1261/rna.078709.121 (PMC8370741; doi:10.1261/rna.078709.121)
Supplement: Supplemental Material [file supp_078709.121_Supplemental_Figures.pptx]

## Slide 1
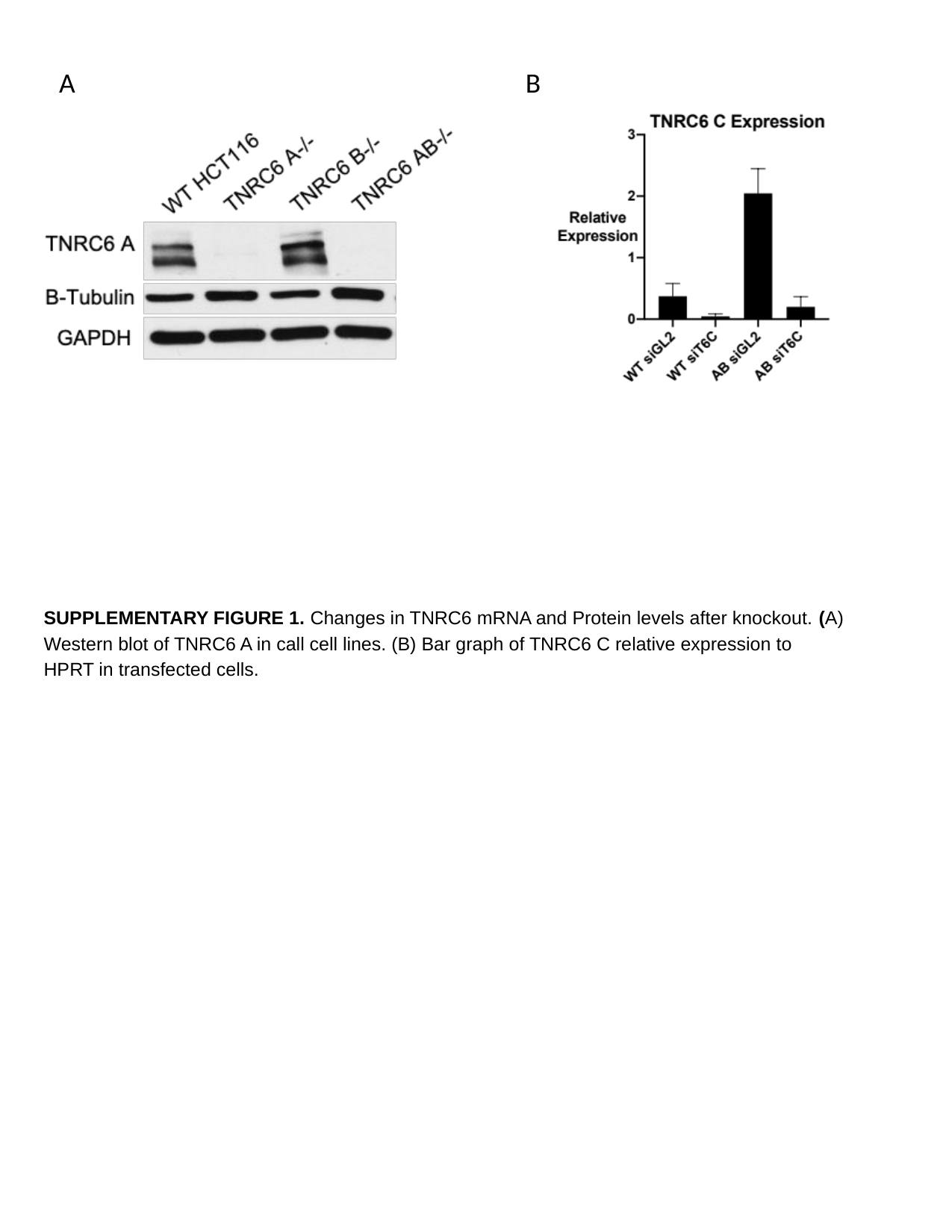

A
B
SUPPLEMENTARY FIGURE 1. Changes in TNRC6 mRNA and Protein levels after knockout. (A) Western blot of TNRC6 A in call cell lines. (B) Bar graph of TNRC6 C relative expression to HPRT in transfected cells.

## Slide 2
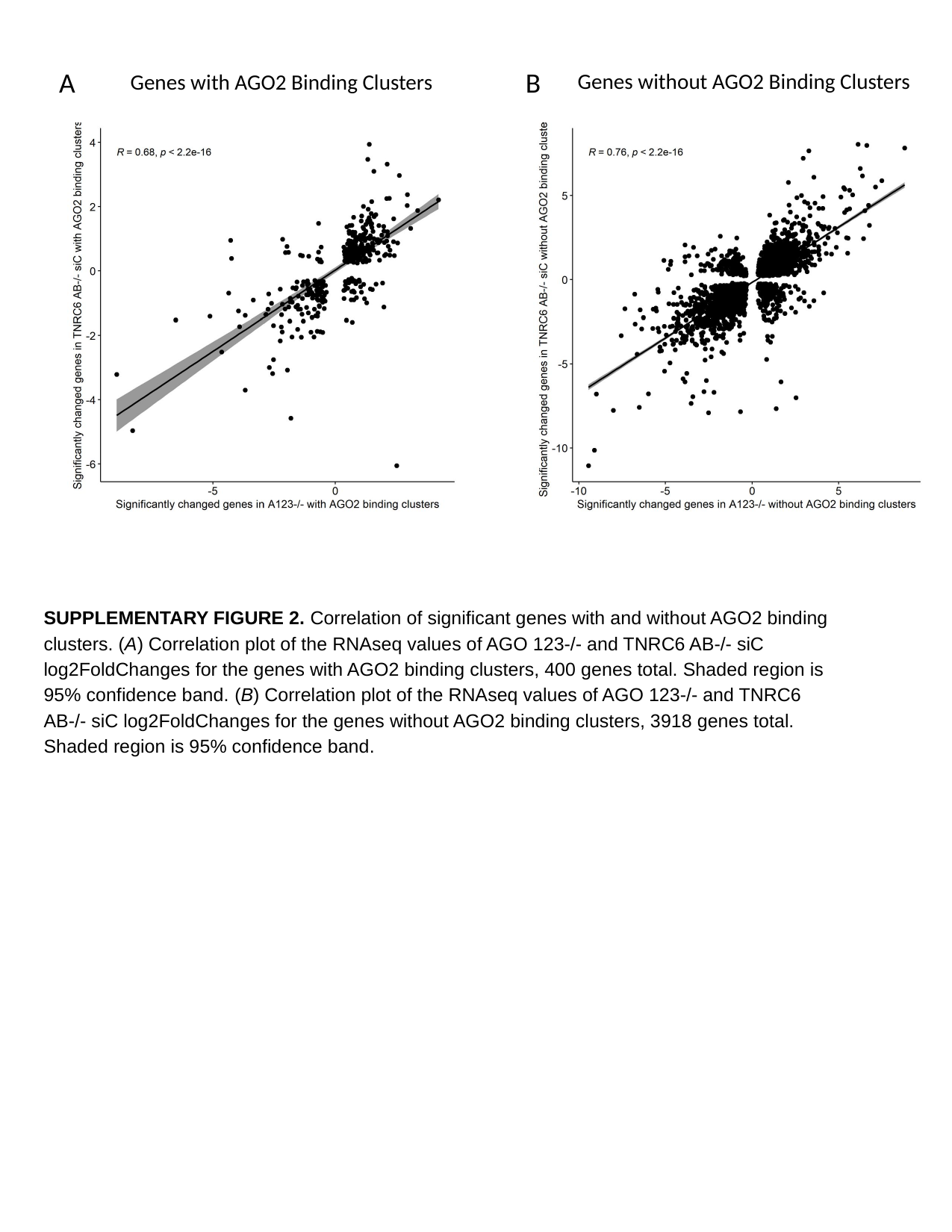

A
B
Genes without AGO2 Binding Clusters
Genes with AGO2 Binding Clusters
SUPPLEMENTARY FIGURE 2. Correlation of significant genes with and without AGO2 binding clusters. (A) Correlation plot of the RNAseq values of AGO 123-/- and TNRC6 AB-/- siC log2FoldChanges for the genes with AGO2 binding clusters, 400 genes total. Shaded region is 95% confidence band. (B) Correlation plot of the RNAseq values of AGO 123-/- and TNRC6 AB-/- siC log2FoldChanges for the genes without AGO2 binding clusters, 3918 genes total. Shaded region is 95% confidence band.

## Slide 3
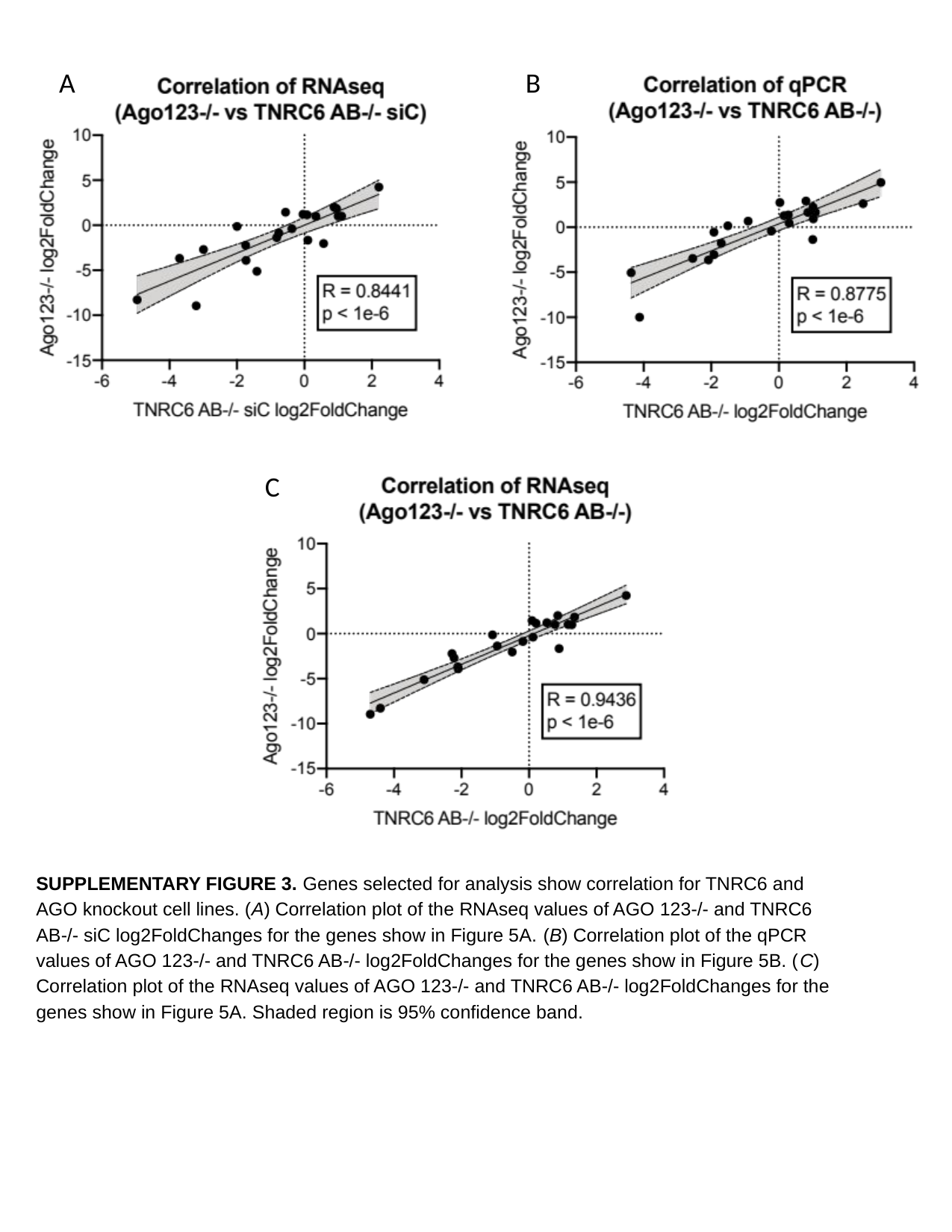

A
B
C
SUPPLEMENTARY FIGURE 3. Genes selected for analysis show correlation for TNRC6 and AGO knockout cell lines. (A) Correlation plot of the RNAseq values of AGO 123-/- and TNRC6 AB-/- siC log2FoldChanges for the genes show in Figure 5A. (B) Correlation plot of the qPCR values of AGO 123-/- and TNRC6 AB-/- log2FoldChanges for the genes show in Figure 5B. (C) Correlation plot of the RNAseq values of AGO 123-/- and TNRC6 AB-/- log2FoldChanges for the genes show in Figure 5A. Shaded region is 95% confidence band.

## Slide 4
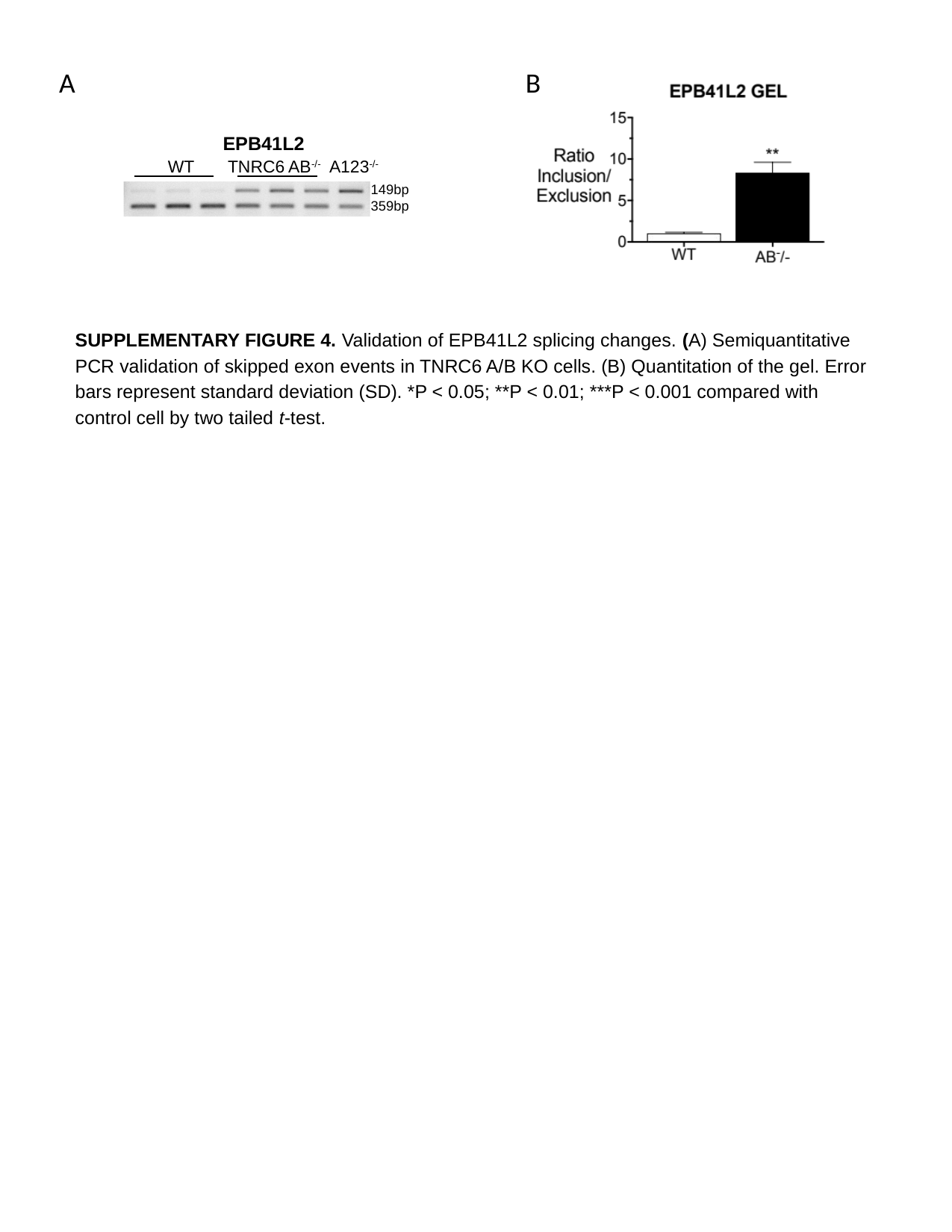

A
B
EPB41L2
WT TNRC6 AB-/- A123-/-
149bp
359bp
SUPPLEMENTARY FIGURE 4. Validation of EPB41L2 splicing changes. (A) Semiquantitative PCR validation of skipped exon events in TNRC6 A/B KO cells. (B) Quantitation of the gel. Error bars represent standard deviation (SD). *P < 0.05; **P < 0.01; ***P < 0.001 compared with control cell by two tailed t-test.

## Slide 5
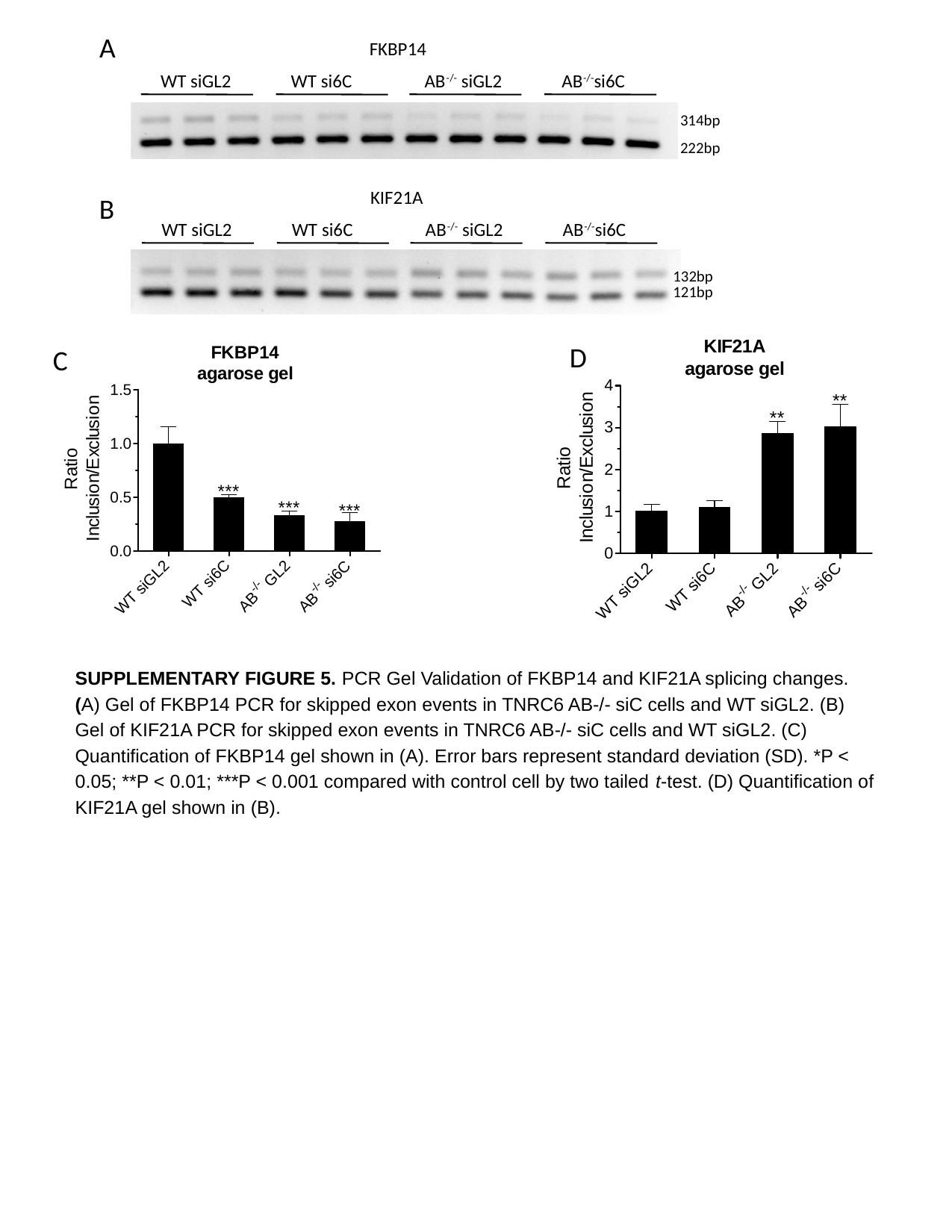

A
FKBP14
 WT siGL2 WT si6C AB-/- siGL2 AB-/-si6C
314bp
222bp
KIF21A
 WT siGL2 WT si6C AB-/- siGL2 AB-/-si6C
132bp
121bp
B
D
C
SUPPLEMENTARY FIGURE 5. PCR Gel Validation of FKBP14 and KIF21A splicing changes. (A) Gel of FKBP14 PCR for skipped exon events in TNRC6 AB-/- siC cells and WT siGL2. (B) Gel of KIF21A PCR for skipped exon events in TNRC6 AB-/- siC cells and WT siGL2. (C) Quantification of FKBP14 gel shown in (A). Error bars represent standard deviation (SD). *P < 0.05; **P < 0.01; ***P < 0.001 compared with control cell by two tailed t-test. (D) Quantification of KIF21A gel shown in (B).

## Slide 6
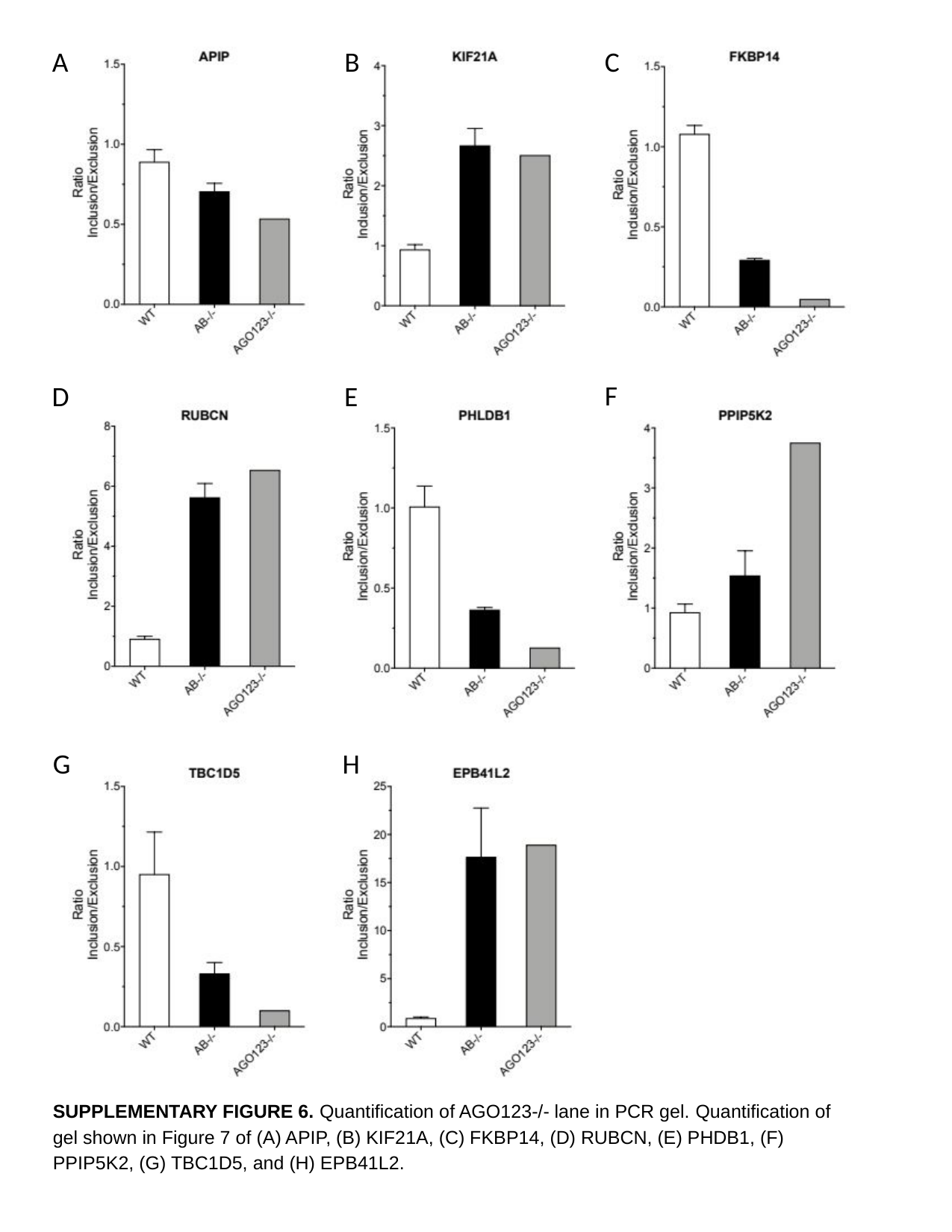

A
B
C
F
D
E
G
H
SUPPLEMENTARY FIGURE 6. Quantification of AGO123-/- lane in PCR gel. Quantification of gel shown in Figure 7 of (A) APIP, (B) KIF21A, (C) FKBP14, (D) RUBCN, (E) PHDB1, (F) PPIP5K2, (G) TBC1D5, and (H) EPB41L2.

## Slide 7
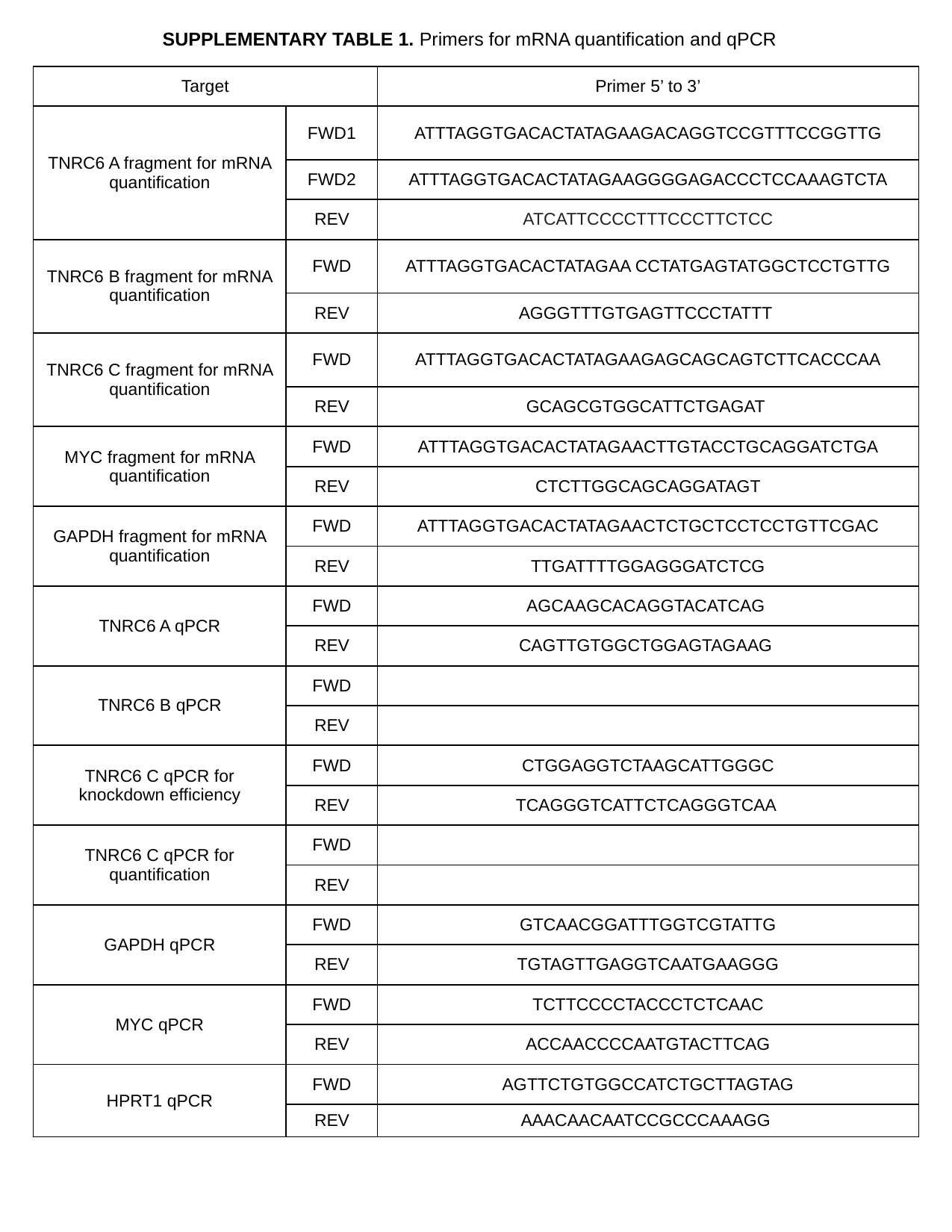

SUPPLEMENTARY TABLE 1. Primers for mRNA quantification and qPCR
| Target | | Primer 5’ to 3’ |
| --- | --- | --- |
| TNRC6 A fragment for mRNA quantification | FWD1 | ATTTAGGTGACACTATAGAAGACAGGTCCGTTTCCGGTTG |
| TNRC6 A fragment for mRNA quantification | FWD2 | ATTTAGGTGACACTATAGAAGGGGAGACCCTCCAAAGTCTA |
| TNRC6 A fragment for mRNA quantification | REV | ATCATTCCCCTTTCCCTTCTCC |
| TNRC6 B fragment for mRNA quantification | FWD | ATTTAGGTGACACTATAGAA CCTATGAGTATGGCTCCTGTTG |
| TNRC6 B fragment for mRNA quantification | REV | AGGGTTTGTGAGTTCCCTATTT |
| TNRC6 C fragment for mRNA quantification | FWD | ATTTAGGTGACACTATAGAAGAGCAGCAGTCTTCACCCAA |
| | REV | GCAGCGTGGCATTCTGAGAT |
| MYC fragment for mRNA quantification | FWD | ATTTAGGTGACACTATAGAACTTGTACCTGCAGGATCTGA |
| | REV | CTCTTGGCAGCAGGATAGT |
| GAPDH fragment for mRNA quantification | FWD | ATTTAGGTGACACTATAGAACTCTGCTCCTCCTGTTCGAC |
| | REV | TTGATTTTGGAGGGATCTCG |
| TNRC6 A qPCR | FWD | AGCAAGCACAGGTACATCAG |
| | REV | CAGTTGTGGCTGGAGTAGAAG |
| TNRC6 B qPCR | FWD | |
| | REV | |
| TNRC6 C qPCR for knockdown efficiency | FWD | CTGGAGGTCTAAGCATTGGGC |
| | REV | TCAGGGTCATTCTCAGGGTCAA |
| TNRC6 C qPCR for quantification | FWD | |
| | REV | |
| GAPDH qPCR | FWD | GTCAACGGATTTGGTCGTATTG |
| | REV | TGTAGTTGAGGTCAATGAAGGG |
| MYC qPCR | FWD | TCTTCCCCTACCCTCTCAAC |
| | REV | ACCAACCCCAATGTACTTCAG |
| HPRT1 qPCR | FWD | AGTTCTGTGGCCATCTGCTTAGTAG |
| | REV | AAACAACAATCCGCCCAAAGG |

## Slide 8
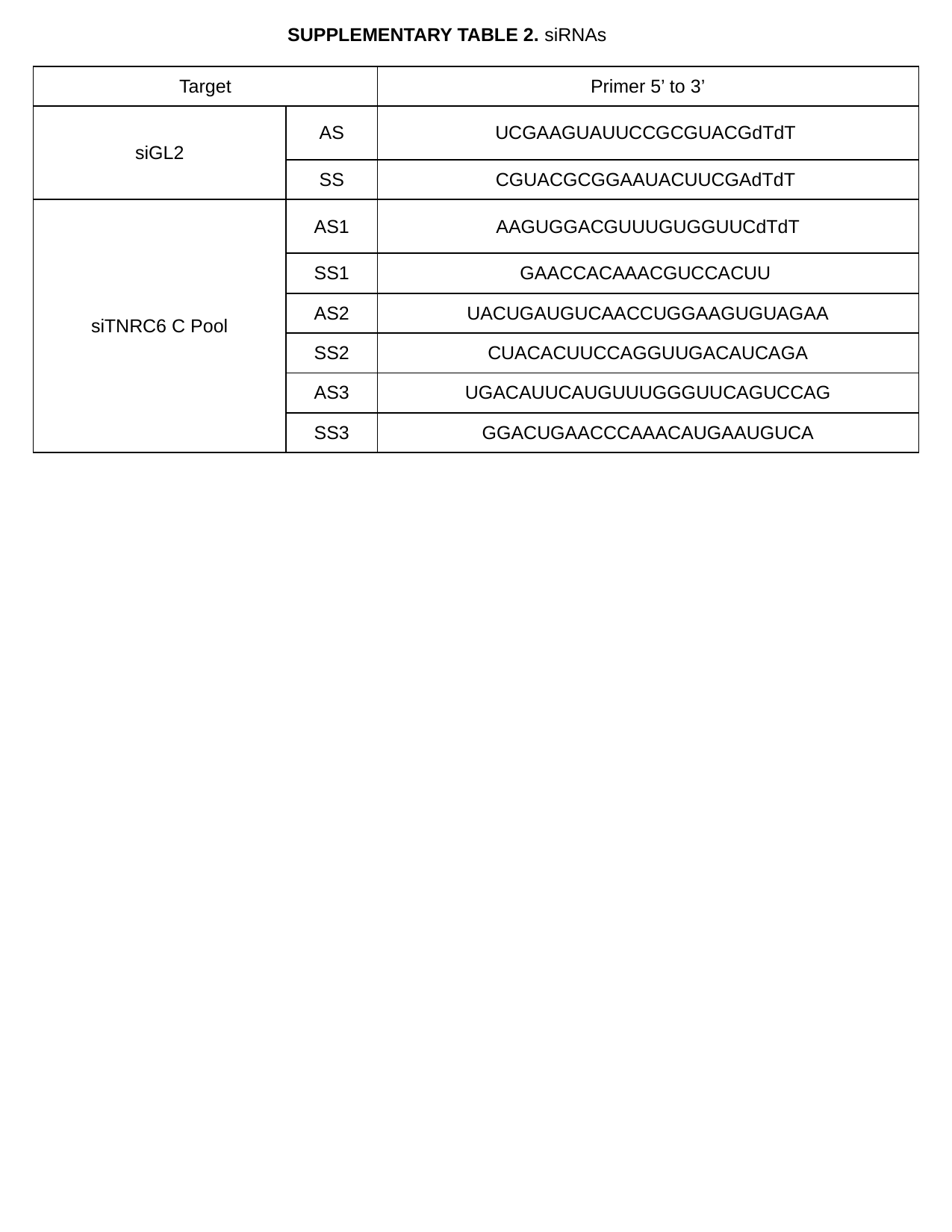

SUPPLEMENTARY TABLE 2. siRNAs
| Target | | Primer 5’ to 3’ |
| --- | --- | --- |
| siGL2 | AS | UCGAAGUAUUCCGCGUACGdTdT |
| TNRC6 B fragment for mRNA quantification | SS | CGUACGCGGAAUACUUCGAdTdT |
| siTNRC6 C Pool | AS1 | AAGUGGACGUUUGUGGUUCdTdT |
| | SS1 | GAACCACAAACGUCCACUU |
| MYC fragment for mRNA quantification | AS2 | UACUGAUGUCAACCUGGAAGUGUAGAA |
| | SS2 | CUACACUUCCAGGUUGACAUCAGA |
| GAPDH fragment for mRNA quantification | AS3 | UGACAUUCAUGUUUGGGUUCAGUCCAG |
| | SS3 | GGACUGAACCCAAACAUGAAUGUCA |

## Slide 9
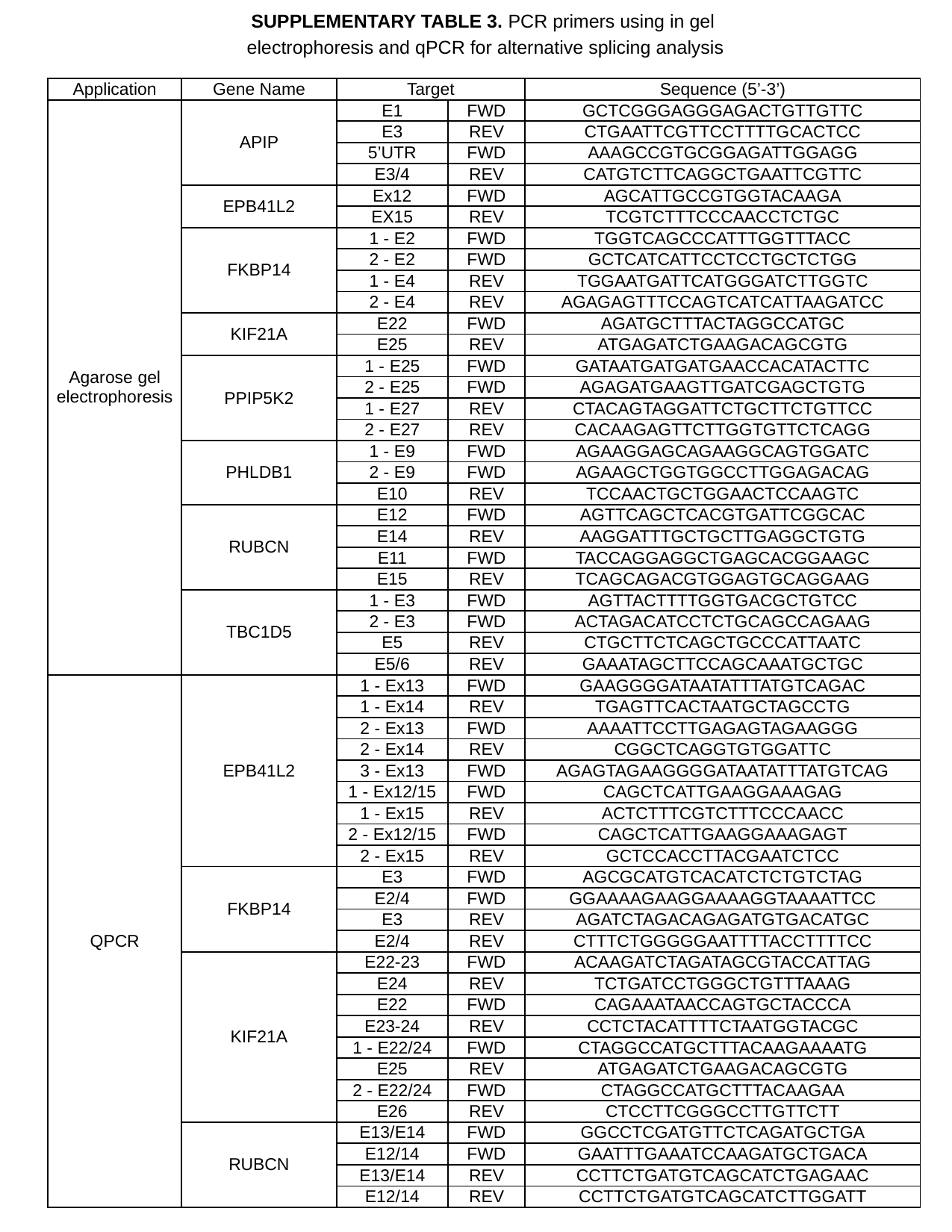

SUPPLEMENTARY TABLE 3. PCR primers using in gel
electrophoresis and qPCR for alternative splicing analysis
| Application | Gene Name | Target | | Sequence (5’-3’) |
| --- | --- | --- | --- | --- |
| Agarose gel electrophoresis | APIP | E1 | FWD | GCTCGGGAGGGAGACTGTTGTTC |
| | | E3 | REV | CTGAATTCGTTCCTTTTGCACTCC |
| | | 5’UTR | FWD | AAAGCCGTGCGGAGATTGGAGG |
| | | E3/4 | REV | CATGTCTTCAGGCTGAATTCGTTC |
| | EPB41L2 | Ex12 | FWD | AGCATTGCCGTGGTACAAGA |
| | | EX15 | REV | TCGTCTTTCCCAACCTCTGC |
| | FKBP14 | 1 - E2 | FWD | TGGTCAGCCCATTTGGTTTACC |
| | | 2 - E2 | FWD | GCTCATCATTCCTCCTGCTCTGG |
| | | 1 - E4 | REV | TGGAATGATTCATGGGATCTTGGTC |
| | | 2 - E4 | REV | AGAGAGTTTCCAGTCATCATTAAGATCC |
| | KIF21A | E22 | FWD | AGATGCTTTACTAGGCCATGC |
| | | E25 | REV | ATGAGATCTGAAGACAGCGTG |
| | PPIP5K2 | 1 - E25 | FWD | GATAATGATGATGAACCACATACTTC |
| | | 2 - E25 | FWD | AGAGATGAAGTTGATCGAGCTGTG |
| | | 1 - E27 | REV | CTACAGTAGGATTCTGCTTCTGTTCC |
| | | 2 - E27 | REV | CACAAGAGTTCTTGGTGTTCTCAGG |
| | PHLDB1 | 1 - E9 | FWD | AGAAGGAGCAGAAGGCAGTGGATC |
| | | 2 - E9 | FWD | AGAAGCTGGTGGCCTTGGAGACAG |
| | | E10 | REV | TCCAACTGCTGGAACTCCAAGTC |
| | RUBCN | E12 | FWD | AGTTCAGCTCACGTGATTCGGCAC |
| | | E14 | REV | AAGGATTTGCTGCTTGAGGCTGTG |
| | | E11 | FWD | TACCAGGAGGCTGAGCACGGAAGC |
| | | E15 | REV | TCAGCAGACGTGGAGTGCAGGAAG |
| | TBC1D5 | 1 - E3 | FWD | AGTTACTTTTGGTGACGCTGTCC |
| | | 2 - E3 | FWD | ACTAGACATCCTCTGCAGCCAGAAG |
| | | E5 | REV | CTGCTTCTCAGCTGCCCATTAATC |
| | | E5/6 | REV | GAAATAGCTTCCAGCAAATGCTGC |
| QPCR | EPB41L2 | 1 - Ex13 | FWD | GAAGGGGATAATATTTATGTCAGAC |
| | | 1 - Ex14 | REV | TGAGTTCACTAATGCTAGCCTG |
| | | 2 - Ex13 | FWD | AAAATTCCTTGAGAGTAGAAGGG |
| | | 2 - Ex14 | REV | CGGCTCAGGTGTGGATTC |
| | | 3 - Ex13 | FWD | AGAGTAGAAGGGGATAATATTTATGTCAG |
| | | 1 - Ex12/15 | FWD | CAGCTCATTGAAGGAAAGAG |
| | | 1 - Ex15 | REV | ACTCTTTCGTCTTTCCCAACC |
| | | 2 - Ex12/15 | FWD | CAGCTCATTGAAGGAAAGAGT |
| | | 2 - Ex15 | REV | GCTCCACCTTACGAATCTCC |
| | FKBP14 | E3 | FWD | AGCGCATGTCACATCTCTGTCTAG |
| | | E2/4 | FWD | GGAAAAGAAGGAAAAGGTAAAATTCC |
| | | E3 | REV | AGATCTAGACAGAGATGTGACATGC |
| | | E2/4 | REV | CTTTCTGGGGGAATTTTACCTTTTCC |
| | KIF21A | E22-23 | FWD | ACAAGATCTAGATAGCGTACCATTAG |
| | | E24 | REV | TCTGATCCTGGGCTGTTTAAAG |
| | | E22 | FWD | CAGAAATAACCAGTGCTACCCA |
| | | E23-24 | REV | CCTCTACATTTTCTAATGGTACGC |
| | | 1 - E22/24 | FWD | CTAGGCCATGCTTTACAAGAAAATG |
| | | E25 | REV | ATGAGATCTGAAGACAGCGTG |
| | | 2 - E22/24 | FWD | CTAGGCCATGCTTTACAAGAA |
| | | E26 | REV | CTCCTTCGGGCCTTGTTCTT |
| | RUBCN | E13/E14 | FWD | GGCCTCGATGTTCTCAGATGCTGA |
| | | E12/14 | FWD | GAATTTGAAATCCAAGATGCTGACA |
| | | E13/E14 | REV | CCTTCTGATGTCAGCATCTGAGAAC |
| | | E12/14 | REV | CCTTCTGATGTCAGCATCTTGGATT |
