# Supplementary material for: Impact of scaffolding protein TNRC6 paralogs on gene expression and splicing
Source: RNA. 2021 Sep;27(9):1004–16. doi: 10.1261/rna.078709.121 (PMC8370741; doi:10.1261/rna.078709.121)
Supplement: Supplemental Material [file supp_078709.121_Supplemental_Figure_Legends.docx]

**SUPPLEMENTARY FIGURE 1.** Changes in TNRC6 mRNA and Protein levels after knockout. **(**A) Western blot of TNRC6 A in call cell lines. (B) Bar graph of TNRC6 C relative expression to HPRT in transfected cells.

**SUPPLEMENTARY FIGURE 2.** (*A*) Correlation plot of the RNAseq values of AGO 123-/- and TNRC6 AB-/- siC log2FoldChanges for the genes with AGO2 binding clusters, 400 genes total. Shaded region is 95% confidence band. (*B*) Correlation plot of the RNAseq values of AGO 123-/- and TNRC6 AB-/- siC log2FoldChanges for the genes without AGO2 binding clusters, 3918 genes total. Shaded region is 95% confidence band.

**SUPPLEMENTARY FIGURE 3.** (A) Correlation plot of the RNAseq values of AGO 123-/- and TNRC6 AB-/- siC log2FoldChanges for the genes show in Figure 5A. (B) Correlation plot of the qPCR values of AGO 123-/- and TNRC6 AB-/- log2FoldChanges for the genes show in Figure 5B. (C) Correlation plot of the RNAseq values of AGO 123-/- and TNRC6 AB-/- log2FoldChanges for the genes show in Figure 5A. Shaded region is 95% confidence band.

**SUPPLEMENTARY FIGURE 4.** Validation of EPB41L2 splicing changes. **(**A) Semiquantitative PCR validation of skipped exon events in TNRC6 A/B KO cells. (B) Quantitation of the gel. Error bars represent standard deviation (SD). *P < 0.05; **P < 0.01; ***P < 0.001 compared with control cell by two tailed *t*-test.

**SUPPLEMENTARY FIGURE 5.** PCR Gel Validation of FKBP14 and KIF21A splicing changes. **(**A) gel of FKBP14 PCR for skipped exon events in TNRC6 AB-/- siC cells and WT siGL2. (B) gel of KIF21A PCR for skipped exon events in TNRC6 AB-/- siC cells and WT siGL2. (C) Quantification of FKBP14 gel shown in (A). Error bars represent standard deviation (SD). *P < 0.05; **P < 0.01; ***P < 0.001 compared with control cell by two tailed *t*-test. (D) Quantification of KIF21A gel shown in (B).

**SUPPLEMENTARY FIGURE 6.** Quantification of AGO123-/- lane in PCR gel. Quantification of gel shown in Figure 7 of (A) APIP, (B) KIF21A, (C) FKBP14, (D) RUBCN, (E) PHDB1, (F) PPIP5K2, (G) TBC1D5, and (H) EPB41L2.
